# Soft ionization by chemical reaction in transfer—high-resolution mass spectrometry for clinical exhaled breath profiling

**DOI:** 10.1007/s00216-026-06425-1

**Published:** 2026-03-15

**Authors:** Camille Roquencourt, Elodie Lamy, Nicolas Hunzinger, Hélène Salvator, Philippe Devillier, Emmanuelle Bardin, Stanislas Grassin-Delyle

**Affiliations:** 1 Hôpital Foch, Exhalomics®, 40, Rue Worth, 92150 Suresnes, France; 2 Institut Hospitalo-Universitaire Comprehensive SEPSIS Center, Paris-Saclay University, Saclay, France; 3 Université Paris-Saclay, UVSQ, INSERM, Infection et Inflammation (2I), U1173, Département de Biotechnologie de la Santé, Unité Technologies pour la Santé et le Médicament, Montigny Le Bretonneux, France; 4 Hôpital Foch, Service de Pneumologie, Suresnes, France; 5 Université Paris-Saclay, UVSQ, UFR Simone Veil-Santé, VIM-Suresnes, UMR0892, Suresnes, France; 6 Université Paris Cité, INSERM U1151, CNRS UMR8253, Institut Necker Enfants Malades, Paris, France

**Keywords:** Breathomics, Real-time mass spectrometry, Volatile organic compounds, Soft ionization by chemical reaction in transfer

## Abstract

Exhaled breath analysis represents a rapid and non-invasive strategy for detecting volatile organic compounds (VOCs) that mirror human metabolic activity and pathological processes. The soft ionization by chemical reaction in transfer (SICRIT) interface, when integrated with high-resolution mass spectrometry (HRMS), provides a flexible and powerful alternative to conventional analytical techniques for breath analysis. This study evaluated feasibility, analytical reproducibility, and chemical coverage of SICRIT-HRMS for clinical exhaled breath analysis. Signal processing included feature alignment, filtering and compound annotation through the Human Metabolome Database (HMDB) and the Human Breathomics Database (HBDB). In 40 healthy volunteers, SICRIT-HRMS reproducibly detected 604 spectral features (80% matching HMDB, 56% HBDB), including alkanes (oxidative stress markers) and semi-volatile amino acids (likely from airway microdroplets). Median reproducibility across exhalations was 13%, and cosine similarity between VOC profiles reached 0.98 within individuals. Pathway enrichment analysis revealed a significant contribution of amino-acid and lipid metabolism. Twenty-five VOCs were associated with gender and six with fasting duration. SICRIT-HRMS enables high-resolution, real-time profiling of the human breath metabolome with strong reproducibility and broad chemical coverage beyond classical VOCs. Its unique capabilities position it as a comprehensive tool for breathomics research and future translational diagnostic applications. ClinicalTrials.gov Identifier: NCT06020521.

**Supplementary Information:**

The online version contains supplementary material available at 10.1007/s00216-026-06425-1.

## Introduction

Breath analysis offers a non-invasive and fast approach to medical diagnostics. It enables the detection of volatile organic compounds (VOCs) in exhaled breath, molecules that reflect physiological and pathological processes [1, 2]. These VOCs can serve as biomarkers for a wide range of diseases. This method holds promise for early detection, monitoring of chronic conditions, and personalized treatment, meeting the growing demand for patient-friendly, real-time diagnostic tools [3, 4].

The most widely used method for exhaled breath analysis is gas chromatography coupled with electron ionization mass spectrometry (GC–MS) [5, 6]. This setup provides both retention times and rich fragmentation spectra, facilitating VOC annotation through matching with spectral libraries [7]. However, GC–MS-based breath analysis requires the use of intermediate breath collection devices (e.g., Tedlar® bags, sorbent tubes) [8], which may introduce analytical bias and variability in the absence of robust standardization methods. The analysis is time-consuming, and factors such as sample collection, storage, compound stability, and analytical blanks are critical to avoid detecting contaminant VOCs from various sources [9–12].

To overcome this limitation, online approaches enabling real-time analysis may be used [13]. The most widely used rely on soft ionization methods, such as proton-transfer reaction mass spectrometry (PTR-MS) and selected-ion flow-tube mass spectrometry (SIFT-MS) [14–18]. Both techniques use reagent ions (H_3_O^+^, NO^+^, O_2_^+^) to produce molecular adducts, although PTR-MS offers superior sensitivity (reaching parts-per-trillion (ppt) levels), due to the specific type of drift tube used between the ionization source and the analyzer [8, 13].

Atmospheric pressure ionization techniques such as atmospheric pressure chemical ionization (APCI) [19] and secondary electrospray ionization (SESI) [19] have recently been applied to real-time breath analysis [13, 20]. These soft ionization methods produce molecular ions (e.g., [M + H]⁺, [M + NH₄]⁺) in both positive and negative modes with minimal in-source fragmentation and they can be coupled to high-resolution mass spectrometry (HRMS) to separate isobaric compounds based on exact mass [21], with already some applications in breath analysis [22]. A new ambient ionization mass spectrometry method, soft ionization by chemical reaction in transfer (SICRIT), recently emerged with potential applications for breath analysis. This technique uses a cold plasma containing a mixture of ions, such as O₂⁺, H₃O⁺, O₂⁻ and NO₂^−^ and enables the generation of various molecular adducts (e.g., M^+^, [M + H]^+^, [M + NH_4_]^+^), which may be detected by HRMS in positive or negative ionization modes [23], potentially increasing the chemical diversity in the detected VOCs. To date, the feasibility of using SICRIT for breath analysis has been demonstrated in a proof-of-concept study involving two volunteers [24], but information on its analytical performance in a clinical research setup is lacking. The objective of the present study was to evaluate the feasibility, analytical reproducibility, chemical coverage, and quality parameters of breath analysis with SICRIT in a well-characterized healthy cohort, for subsequent application in clinical research for biomarker discovery.

## Materials and methods

### Study design and participants

We conducted a prospective, open-label study at a university hospital. The study protocol was registered (VOC-COMPARE, NCT06020521, 2023–07–08) and approved by an independent ethics committee (Comité de Protection des Personnes Sud-Est VI). Written informed consent was obtained from all participants. The main inclusion criterion was healthy adult volunteers, while exclusion criteria included pregnancy, any chronic medical condition, and active smoking. The study consisted of a single visit during which breath analysis was performed using multiple techniques; the results of the SICRIT-HRMS analysis are reported herein. Demographic data, time since last meal, and any current medication use were recorded for each participant.

### Study measurements and procedures using SICRIT-HRMS

**Fig. 1:**
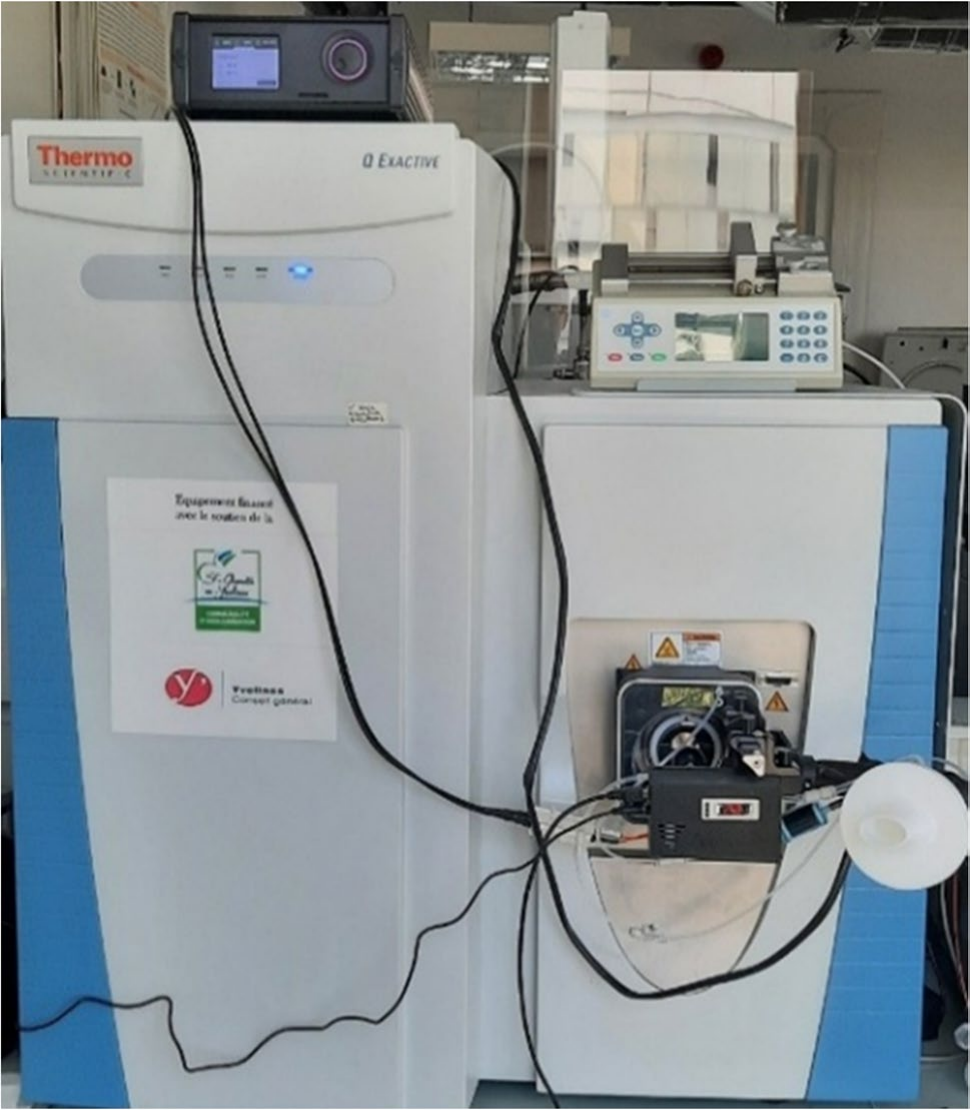
Photograph of the SICRIT ionisation source coupled to a Q Exactive high-resolution mass spectrometer (SICRIT-HRMS), which was employed for the analytical measurements

Analyses were conducted in the morning following a minimum fasting period of three hours. Breath analysis consisted of three consecutive deep inhalations, each followed by an exhalation through a single-use mouthpiece equipped with a filter (MicroGard II, Vyaire Medical GmbH, Hoechberg, Germany). This mouthpiece was connected to the SICRIT® breath analysis module (Plasmion GmbH, Augsburg, Germany), interfaced with the mass spectrometer. Expiratory airflow was monitored in real-time, and participants were instructed to maintain a flow rate as close as possible to 8 L/min. A split breath dilution at a 1:5 ratio was performed using nitrogen (VWR, Fontenay-sous-Bois, France). Background measurements were recorded daily throughout the study. The SICRIT operational settings were as follows: voltage 1600 V, frequency 15,000 Hz, temperature 150 °C. Detection was performed using a Q-Exactive mass spectrometer (ThermoFisher, Courtaboeuf, France) operating in positive ionization mode, with full-scan acquisition in the 50–300 *m/z* range. Sheath and auxiliary gas flow rates were set to 0 and 12 arbitrary units, respectively. The capillary temperature was maintained at 200 °C, and the S-Lens RF level was set to 65 V. A scheme of the setup is shown in Fig. 1.

### Signal processing

Signal processing was performed using an in-house R script, following the conversion of raw data files to the mzML format. The first step of the workflow involved mass axis recalibration using isoprene at *m/z* 69.070 [C_5_H_8_ + H]^+^, and lock masses at *m/z* 149.045 [[Si(CH_3_)_2_O]_2_ + H]^+^ and *m/z* 223.064 [[Si(CH_3_)_2_O]_3_ + H]^+^. Peak detection within each spectrum was performed using the *pracma* R package. Detected *m/z* values were then aligned across samples using the *align* function from the *ptairMS* R package [25], generating temporal intensity traces for each peak over the course of the acquisition. Exhalation phases were identified using isoprene (*m/z* 69.070), a compound consistently present in human breath. Each compound was subsequently quantified as the mean intensity during the three exhalation phases. Lists of detected *m/z* values were further aligned across individuals using the *ptairMS* alignment function. After alignment, we filtered VOCs to retain only those detected in more than 30% of participants and showing significantly higher intensity during exhalation compared to background in over 30% of samples. Missing values were imputed using the probabilistic minimum imputation (MinProb) method via the *imputeLCMD* R package. Isotopes were then identified based on inter-sample correlations, expected abundance ratios based on putative formula annotations, or ratios below 20% when no matching formula was available, and mass differences. They were subsequently removed.

### Feature annotation and pathway analysis

Breath VOCs were putatively annotated by matching their *m/z* values to entries in two open-access online databases, allowing a maximum mass deviation of 10 ppm: the Human Metabolome Database (HMDB, version 5.0) [26] which includes nearly 50,000 compounds, and the Human Breathomics Database (HBDB) [27], which contains 1,034 compounds previously reported in human breath in the literature. These two databases serve complementary purposes: HMDB is the most comprehensive resource for human metabolism, while HBDB provides evidence-based confirmation of metabolite presence in exhaled breath from prior studies. To further enhance coverage, we supplemented these resources with 705 additional compounds identified from a recent updated review of VOCs in exhaled breath [28]. In line with the literature [23], we considered a range of possible adducts for *m/z* matching, including [M + H]^+^, [M]^+^, [M-H]^+^, [M-OH]^+^, and [M + NH_4_]^+^.

Metabolomic pathway analysis was performed using *mummichog* [29] and the MFN database, which predicts biological function based on putative annotations without requiring formal metabolite identification. All metabolites detected by the SICRIT instrument were included as recommended [29] (including VOCs from either breath or ambient air), with the associated *p*-value (breath *vs* air) and the intensity of each feature measured in exhaled breath.

### Reproducibility metrics

To assess various aspects of the reproducibility of SICRIT-HRMS signals in exhaled breath, we employed three distinct metrics. These metrics were calculated for each *m/z* feature and for each individual in whom signal intensities were higher during the exhalation phase than in ambient air. Final results were aggregated using the median:

- Reproducibility across exhalations was assessed for each *m/z* feature using the coefficient of variation (CV) of the mean intensity across the three exhalations.
- Stability within a single exhalation was evaluated by calculating the CV of signal intensity of each *m/z* feature throughout the duration of a single exhalation (CV averaged across the three exhalation).
- Intra-individual similarity of temporal VOC profiles, i.e. the reproducibility of the profile of VOCs exhaled by each individual, was quantified using a cosine similarity matrix, which measures pairwise cosine similarity between vectors, reflecting the degree of similarity between two n-dimensional vectors based on the cosine of the angle between them [30].

We finally evaluated the inter-subject variability by first calculating the CV across patients for each VOC, and then averaging these CVs.

### Statistical analysis

Hierarchical clustering analysis (HCA) was used to identify clusters among the detected VOCs in exhaled breath, employing Pearson correlation as the distance metric and retaining clusters with an average within-group correlation greater than 0.8. Additionally, univariate statistical tests were performed to assess associations between VOCs and demographic variables: the Mann–Whitney rank-sum test was used for categorical factors, and the Spearman correlation test for continuous variables. VOCs with a *p*-value < 0.05 were considered significantly associated. Finally, principal component analysis (PCA) was conducted to visualize and summarize the relationships among multiple VOCs significantly associated with a given demographic factor.

## Results

### Study population

Forty volunteers participated in the study between August 16th and 30th, 2023. The cohort included 23 females and 17 males, with a median age of 32 years [26–38] and a BMI of 22.9 [20.0–24.8] (median [25th-75th percentiles]). Twenty-five participants had fasted for more than 3 h, and 15 had fasted for more than 15 h. None of the participants were undergoing medical treatment or had taken medication in the days prior to sampling. Breath samples were successfully collected from all participants.

### Breath analysis

#### Breath phases and feature detection

**Fig. 2:**
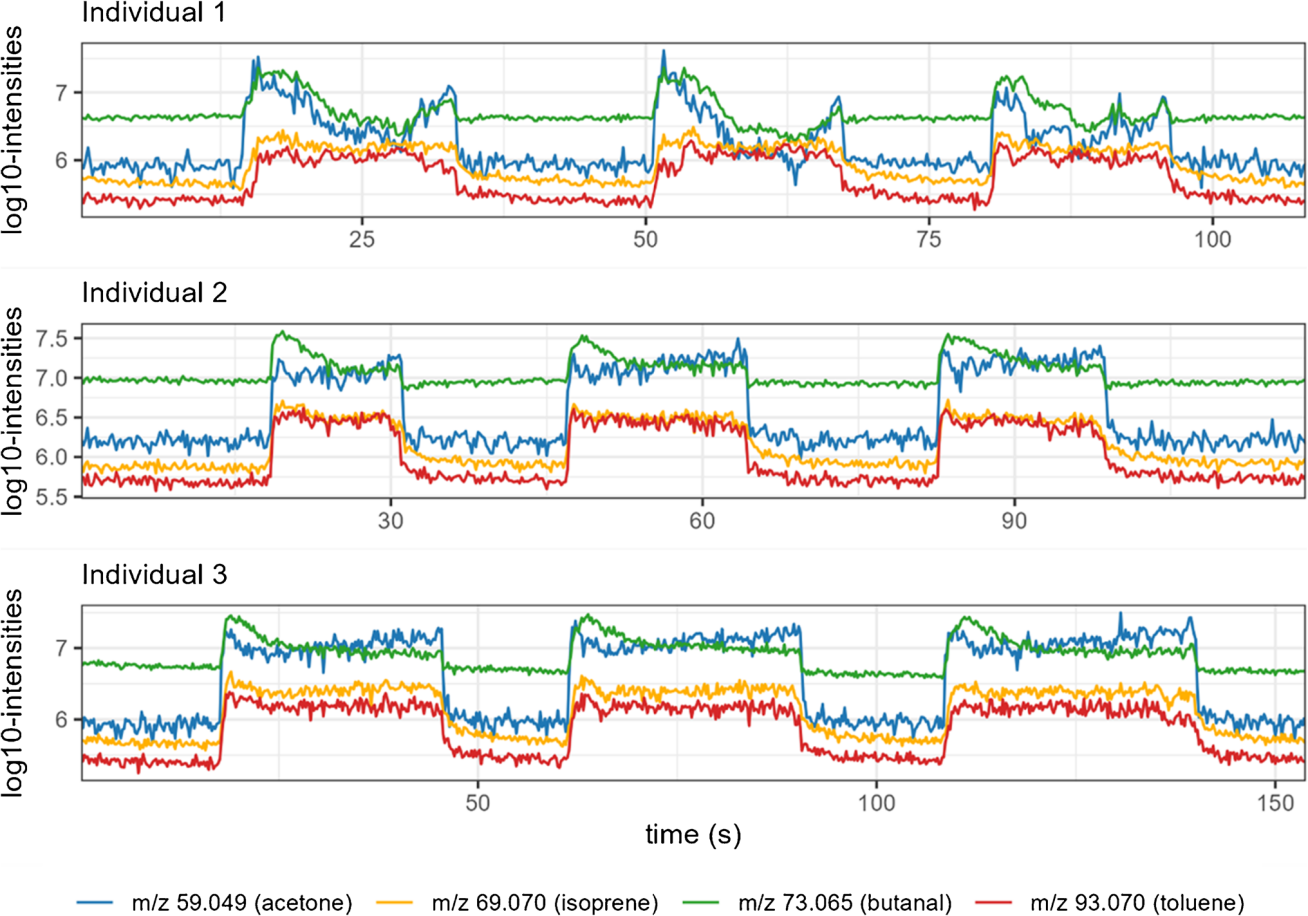
Breath traces showing the dynamics of exhalation phases for four VOCs (with putative annotations) in three representative participants

**Fig. 3:**
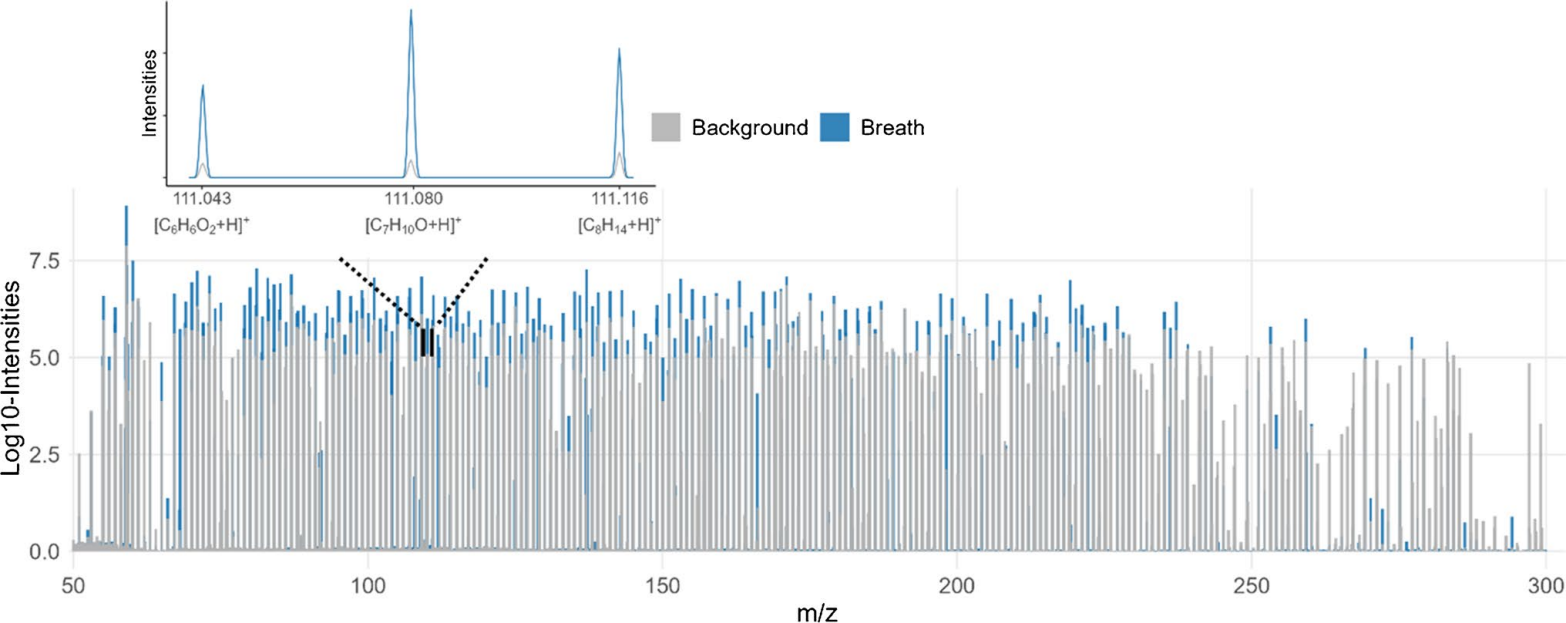
Representative example of superimposed total average spectra corresponding to breath phases and background obtained from the same acquisition, with data binned by nominal mass, with an enlarged view around *m/z* 111 highlighting the mass resolution

Breath phases were accurately identified detected across all three exhalations. Figure 2 presents the signal traces for four *m/z* features during the three exhalation phases in three representative participants. On average, 1,090 *m/z* features were detected per sample, with 59% showing significantly higher abundance during exhalation phases compared to background. Following alignment, filtering, background subtraction, and isotope removal, a total of 604 features were retained within the *m/z* range of 51–294. Of these, 417 features were detected in the breath of all participants. A representative example of superimposed total average spectra corresponding to breath phases and background obtained from the same acquisition is shown in Fig. 3. The average signal-to-noise ratio among breath features was 45. The resolution, calculated using the three calibration peaks (*m/z* 67.069, 149.044, and 223.063), ranged from 75,000 to 130,000.

#### Intra-subject reproducibility

Within-subject median reproducibility across the three exhalation phases was 13% (interquartile range: 7–24%), while the median CV within a single exhalation was 44% (interquartile range: 29–70%). Finally, the cosine similarity between VOC traces ranged from 0.54 to 0.99, with an average median value of 0.98, indicating high signal reproducibility across VOCs within the same individual.

#### Inter-subject reproducibility

The mean CV between individuals was 70%, with a standard deviation of 40%.

#### Feature annotation

When considering all adducts, 80% of the 604 detected compounds matched entries in the Human Metabolome Database, and 56% matched the Human Breathomics Database. This resulted in a total of 2,789 potential compound annotations, including 938 VOCs previously reported in exhaled breath. Detailed results for each adduct match are provided in Table 1. The [M + H]^+^ adduct was the most frequently observed, accounting for 69% of annotated compounds. The 20 most intense breath VOCs in the cohort, along with their [M + H]^+^-based annotations, are listed in Table 2.

**Table 1:** Results of library queries (HMDB, HBDB, and both) for the 604 detected features

Adducts	HMDB		HBDB		Both	
	Features with ≥ 1 match	Corresponding number of putative VOCs	Features with ≥ 1 match	Corresponding number of putative VOCs	Features with ≥ 1 match	Corresponding number of putative VOCs
Total	483 (80%)	2374	339 (56%)	938	491 (81%)	2 789
[M-H]^+^	209 (35%)	609	84 (14%)	200	216 (36%)	673
[M-OH]^+^	266 (44%)	880	98 (16%)	206	271 (45%)	928
[M]^+^	222 (37%)	480	40 (7%)	130	224 (37%)	530
[M + H]^+^	405 (67%)	1 530	220 (36%)	908	418 (69%)	1 893
[M + NH_4_]^+^	163 (27%)	420	55 (9%)	220	168 (28%)	524

Results are expressed as *n* (% of total features)

**Table 2:** The 20 most intense features, including detected isotopes and putative annotations for the [M + H]^+^ adduct, detected in the breath of all 40 participants

*m/z*	*m/z* of isotopes	Median log intensity	Molecular formula	IUPAC name	PubChem IDs	Previously detected in human breath
59.049	60.053 61.054	20.1	C_3_H_6_O	acetone/propanal/prop-2-en-1-ol	180/527/7858	Yes
171.149	172.152	16.5	C_9_H_18_N_2_O	2-amino-3-methyl-1-(pyrrolidin-1-yl)butan-1-one*	4470972	No
87.044	88.047	16.3	C_4_H_6_O_2_	butane-2,3-dione/tetrahydrofuran-2-one/2,3-dihydro-1,4-dioxine/(E)-but-2-enoic acid/acetic acid, ethenyl ester/vinyl acetate/2-butenal/crotonaldehyde	650/7302/136353/637090	Yes
73.065	74.068	16.3	C_4_H_8_O	butanal/2-methylpropanal/butan-2-one/vinyloxyethane/cyclobutanol/ethyleneglycol mono vinyl ester/vinyloxyethanol/tetrahydrofuran	261/6561/6569/8023	Yes
71.049	72.057	16.2	C_4_H_6_O	2-methylprop-2-enal/but-3-en-2-one/2,5-dihydrofuran/2,3-dihydrofuran/(E)-but-2-enal	6562/6570/15570/70934/447466	Yes
101.059	102.063	16.0	C_5_H_8_O_2_	methyl 2-methylprop-2-enoate/ethyl prop-2-enoate/tetrahydropyran-2-one/(E)−2-methylbut-2-enoic acid/(E)-pent-2-enoic acid/methyl (Z)-but-2-enoate/dihydro-5-methyl-2(3H)- furanone/gamma valerolactone/dihydro-4-methyl-2(3H)- furanone/3- methylbutyrolactone/gGlutaraldehyde/2-methyl-2-butenoic acid/2-pentenoic acid	6658/8821/10953/125468/638122/643800	Yes
163.132	164.135 165.127 166.130	16.0	C_8_H_18_O_3_	1-(2-butoxyethoxy)ethanol	41088	Yes
83.049		15.9	C_5_H_6_O	2-methylfuran/3-methylfuran/cyclopent-2-en-1-one	10797/13587/13588	Yes
70.065	71.067	15.9	C_4_H_7_N	2-methylpropanenitrile/butanenitrile/1-pyrroline	6559/8008	Yes
109.101	110.104	15.8	C_8_H_12_	cyclohexene, 4-ethenyl-/4 -vinylcyclohexene/2,3-dimethyl-1,3- cyclohexadiene/1,2-dimethyl-1,4- cyclohexadiene		Yes
81.07	82.073	15.8	C_6_H_8_	cyclohexa-1,3-diene/cyclohexa-1,4-diene/5-methylcyclopenta-1,3-diene/1-methylcyclopenta-1,3-diene/3-methylenecyclopentene/4-methylenecyclopentene/(Z)−3-methylpent-3-en-1-yne/(3Z)-hexa-1,3,5-triene/3-methyl-3-penten-1-yne	11605/12343/25512/66775/136723/556370/5367413/5367415	Yes
170.096	171.099	15.6	C_12_H_11_N	N-phenylaniline/3-phenylaniline	11,87/16717	No
205.143	206.168 207.138	15.6	C_10_H_20_O_4_	1,4,8,11-tetraoxacyclotetradecane	13615995	No
85.065		15.5	C_5_H_8_O	3,4-dihydro-2H-pyran/cyclopentanone/3-methylbut-3-en-2-one/pent-1-en-3-one/3-methylbut-2-enal/(E)-pent-3-en-2-one/(E)−2-methylbut-2-enal/NA/2-ethylacrolein/2- ethacrolein/3-methyl-2-butenal/3-penten-2-one, (E)-	8080/8452/13143/15394/61020/637920/5321950/59270504	Yes
137.132	138.135	15.5	C_10_H_16_	2,2-dimethyl-3-methylene-norbornane/2,6,6-trimethylbicyclo[3.1.1]hept-2-ene/5-isopropyl-2-methyl-cyclohexa-1,3-diene/1-isopropyl-4-methyl-cyclohexa-1,4-diene/1-isopropyl-4-methyl-cyclohexa-1,3-diene/1,7,7-trimethylbicyclo[2.2.1]hept-2-ene/3-isopropyl-6-methylene-cyclohexene/4-isopropylidene-1-methyl-cyclohexene/6,6-dimethyl-2-methylene-norpinane/4-isopropenyl-1-methyl-cyclohexene/3,7,7-trimethylbicyclo[4.1.0]hept-3-ene/7-methyl-3-methylene-octa-1,6-diene/1-isopropyl-4-methylene-cyclohexene/(3E)−3,7-dimethylocta-1,3,6-triene/(3E,5E)−3,7-dimethylocta-1,3,5-triene/cyclohexene, 1-methyl-4-(1- methylethenyl)-, (S)-/(S)-(-/3,7-dimethyl-1,3,6-octatriene/1,4-dimethyl-4- vinylcyclohexene/5-isopropenyl-1-methyl-1- cyclohexene	6616/6654/7460/7461/7462/10047/11142/11463/14896/22311/26049/31253/66841/5281553/6434062	Yes
153.127	154.130	15.4	C_10_H_16_O	1,7,7-trimethylnorbornan-2-one/6-isopropyl-3-methyl-cyclohex-2-en-1-one/adamantan-1-ol/2-hexylfuran/2-(2,2,3-trimethylcyclopent-3-en-1-yl)acetaldehyde/2-isopropyl-5-methyl-cyclohex-2-en-1-one/5-isopropyl-3,3-dimethyl-2-methylene-furan/(-)-carveol/2-cyclohexen-1-ol, 1- methyl-4-(1- methylethenyl)-, trans-/2,3-dehydro-1,8-cineole	2537/6987/64152/77408/98497/107372/586164	Yes
197.153	198.157	15.4	C_12_H_20_O_2_	4-hydroxy-2,6- dodecadienal/linalyl acetate	71340423	Yes
136.021	137.024 138.017	15.3	C_7_H_5_NS	1,3-benzothiazole	7222	Yes
99.044		15.3	C_5_H_6_O_2_	2-furylmethanol/2-methyl-2H-furan-5-one/4-methyl-2H-furan-5-one/3-methyl-2(5H)-furanone	7361/24838276/30945	Yes
67.054		15.3	C_5_H_6_	cyclopenta-1,3-diene/2-methylbut-1-en-3-yne/pent-3-en-1-yne/(E)-pent-3-en-1-yne/2-penten-4-yne/3-penten-1-yne, (Z)-	7612/62323/137090/638083	Yes

*Could also correspond to 3-pentanol, methyl tert-butyl ether or 2-methyl-2-butanol ([M + 2ACN + H]^+^ adduct) or tetraethylurea ([M-H]^−^ ion)

#### Pathway analysis

Five pathways were found to be significant using Mummichog (enrichment *p*-value Fisher < 0.05). These are detailed in Table 3 and presented in Fig. S1, along with their associated *p*-values and enrichment factors. Two pathways were related to amino acids, one to lipids and fatty acids, and one to the tricarboxylic acid cycle. The pathway with the most hits was *Tyrosine metabolism*, which included 22 matched compounds with [M + H]^+^ adducts and 63 with all possible adducts, corresponding to 40% of the total hits described on the pathway. Among the proposed KEGG annotations of metabolites, 49% of the hits are found in HMDB and 17% in HBDB.

**Table 3:** Summary of the Mummichog pathway annotation analysis

Pathway	Class	Pathway total size	Hits	[M + H]^+^ hits	HMDB/HBDB Hit	Enrichment factor	*p*-value (Fisher)
Tyrosine metabolism	Amino acids	160	63	22	26/5	1.93	< 0.001
*de novo* fatty acid biosynthesis	Lipids and fatty acids	106	14	8	13/3	0.65	0.006
Valine, leucine and isoleucine degradation	Amino acids	65	18	11	10/5	1.36	0.009
Butanoate metabolism	TCA cycle	34	18	12	11/4	2.60	0.032

Columns represent the following information: pathway name, associated chemical class, number of features mapped to each pathway, number total of features identified, number of features with an [M + H]^+^ adduct, number of compounds present in the HMDB and/or HBDB databases, and the enrichment factor and *p*-value calculated by the Mummichog algorithm

#### Feature interrelationships and molecular grouping

Hierarchical clustering analysis was applied to the 604 features detected in breath, resulting in 60 groups with an average correlation above 0.8, and 59 unclustered features. The number of features per correlated group ranged from 2 (in 23 of the 60 groups) to 75. This indicates a high degree of information redundancy in the data obtained through SICRIT analysis. Inter-individual variability varied across clusters, with a median coefficient of variation within groups of 66%, ranging from 20 to 216%. The most frequent mass differences between features within the same group corresponded to oxygen (O) (*m/z* 15.995), CH_2_ (*m/z* 14.02) and H_2_ (*m/z* 2.016).

#### Impact of demographic and physiological variables

Finally, the influence of gender, BMI, age, and time since the last meal on the breath profile was assessed. Twenty-five features were significantly associated with gender, six of which were elevated in women; nine of these compounds were annotated in the HBDB. Six features showed significant correlations with fasting duration, with five increasing with longer fasting times. The corresponding PCA score plots built with these significant features are presented in Fig. S2; however, neither plot clearly separates the groups, as substantial overlap was observed. These features are listed in supplementary tables S1 and S2, along with their corresponding HBDB annotations based on the [M + H]^+^ adduct. No significant feature associations were found with age or BMI.

## Discussion

This study represents the first technological validation of SICRIT-HRMS for clinical breath analysis. The primary objectives were to demonstrate feasibility, analytical reproducibility, and chemical coverage in a homogeneous cohort of healthy volunteers. Disease-specific biomarker discovery will require subsequent studies in targeted patient populations. Here, we demonstrate that SICRIT-HRMS enables the real-time detection of a large number of features with a high resolution, supporting its potential for comprehensive breath metabolomics. This technique combines several key advantages: i) real-time, direct analysis of native breath samples without intermediate; ii) plasma ionization, producing a wide variety of molecular adducts and expanding chemical coverage [26], and iii) coupling to high-resolution mass spectrometry, which enables the resolution of isobaric compounds and accurate *m/z*-based annotation.

From a technical standpoint, intra-subject reproducibility was satisfactory, with a median coefficient of variation of 13% across exhalations. Variability within a single exhalation was higher (44%), most likely reflecting physiological fluctuations in expiratory flow, a recognised challenge in breath analysis when flow control is not implemented. Achieving a consistent airflow level proved difficult even in healthy volunteers. Although such variability remains acceptable for exploratory research, it could represent a limitation in studies involving patients with compromised respiratory function. Moreover, like SESI-MS, the real-time SICRIT-HRMS approach does not yield absolute concentrations of VOCs. In contrast, techniques such as PTR-MS can estimate theoretical absolute concentrations based on known reaction rate coefficients and have reported relative standard deviations below 5% for compounds such as acetone [17]. The current SICRIT-HRMS data processing workflow therefore relies on relative signal intensities, indicating that methodological refinement could enhance analytical stability and enable quantitative interpretation.

In our cohort of 40 healthy volunteers, SICRIT-HRMS reproducibly detected 604 features, including 487 that were shared across all participants. Several studies have also characterized the exhaled breath of healthy individuals using alternative technologies and the results are summarized in Table 4. In a TD‑GC‑MS study of 90 individuals from a heterogeneous population, 1,471 signals were detected in total, of which 585 VOCs were significantly more abundant in exhaled breath than in background air and 148 were successfully annotated [31]. Among these 148 annotated VOCs, 83 may form [M + H]^+^ adducts matching *m/z* values (within a 10 ppm deviation) detected in our SICRIT-HRMS dataset. The majority of these overlapping VOCs were organo-oxygen compounds (19), benzenic derivates (10), and unsaturated hydrocarbons (11). Other GC‑MS and GC × GC‑MS studies in healthy subjects typically have reported from a few dozen to a few hundred annotated compounds (for example, 67 targeted VOCs in 28 volunteers with NTD‑GC‑MS, 65 annotated VOCs in a TD‑GC × GC‑qMS/FID study of 7 subjects, and 32–35 compounds in a cohort of 158 participants analyzed by SPME‑GC‑MS, as detailed in Table 4), with the total number of detected features in some series exceeding 2,000 signals [32]. In the field of real‑time mass spectrometry, a SESI‑HRMS study in 31 healthy volunteers reported 574 features, including 48 present in at least 20% of participants and confirmed by MS/MS analysis, 30 of which have corresponding *m/z* features in our SICRIT‑HRMS dataset [33]. Other SESI‑HRMS work in children (56 healthy controls) has detected 2,315 features and 134 annotated compounds, while PTR‑MS and PTR‑TOF‑MS studies in cohorts ranging from 40 to over 500 healthy volunteers have detected between 9 (targeted) VOCs and up to 500 features, with 7 to 32 compounds annotated depending on the instrumental configuration [34]. Within this landscape, the detection of 604 reproducible features in 40 healthy volunteers with SICRIT‑HRMS, 80% of which have at least one HMDB annotation and 56% at least one HBDB annotation, places this method on par with established techniques in terms of chemical coverage and annotation capacity, while demonstrating substantial overlap with GC‑MS, GC × GC‑MS, SESI‑HRMS, and PTR‑MS breath profiles in healthy subjects. These overlaps support the relevance and consistency of SICRIT-HRMS in capturing a representative subset of the human breath volatilome.

**Table 4:** Summary of breath analysis studies in healthy volunteers

Study	Year	*n*	Method		Total number of features	Number of annotated VOCs	Reference
Pauling et al.	1971	Unknown	OFFLINE	GC–MS	~ 250	-	[47]
Phillips et al.	1997	20		TD-GC–MS	150–200 per sample; 1259 in the whole cohort	~ 100	[48]
Phillips et al.	1999	50		TD-GC–MS	157–241 per sample; 3481 in the whole cohort	~ 100	[49]
Mochalski et al.	2013	28		NTD-GC–MS (targeted)	67	67	[50]
Yamanaka et al.	2021	7		TD‑GC × GC‑qMS/FID	> 100	65	[51]
Arulvasan et al.	2024	90 including 24 with chronic diseases		TD‑GC–MS	1471	148	[31]
Schulz et al.	2025	158		SPME–GC–MS	2707	32–35	[32]
Sasiene et al.	2024	31	ONLINE	SESI-HRMS	574	48	[33]
Weber et al.*	2023	56 children		SESI-HRMS	2315	134	[34]
Moser et al.	2005	344 persons attending a public health fair		PTR‑MS (H₃O⁺)	179	15	[52]
Jia et al.	2024	504		PTR‑MS (H₃O⁺)	9 (targeted)	9	[53]
Mustafina et al.*	2025	254		PTR‑MS (H₃O⁺)	511	7	[54]
Houssni et al.*	2025	40 children		PTR‑MS (H₃O⁺ + NH₄⁺)	441 (296 H₃O⁺, 145 NH₄⁺)	32 (24 H₃O⁺, 8 NH₄⁺)	[55]

*Studies including healthy volunteers as controls among other patient populations, features were detected in the whole study population; NTD: needle-trap device; SPME: solid-phase microextraction

While exact-mass matching without MS/MS confirmation is inherently putative, we implemented a robust multi-level validation framework to enhance confidence; including database triangulation (features were matched against HMDB (80% hit rate), HBDB (56%), and a literature-derived VOC list, yielding a total of 2,789 potential annotations for 604 features) and hierarchical clustering (60 correlated feature groups (Pearson r > 0.8) with expected mass differences (O: 15.995, CH₂: 14.02, H₂: 2.016) were identified, consistent with expected adduct formation, isotope peaks, neutral losses, or in-source fragmentation [26]). In some cases, shared biological origin may also account for inter-feature correlations. The predominant adduct was [M + H]^+^, followed by [M − OH]^+^, consistent with previous findings on plasma ionization behaviour [23]. While redundancy is expected in direct-injection high-resolution MS data, it highlights the importance of integrating complementary techniques such as GC × GC–MS or MS/MS to enhance the reliability and specificity of compound annotation [6, 35, 36]. Building spectral databases through the infusion of analytical standards may also aid in elucidating the ionization process, thereby facilitating more accurate compound annotation.

A wide array of compounds, notably including alkanes, was identified using the plasma-based SICRIT ionization technique. Alkanes are thought to stem from oxidative stress and lipid peroxidation [37, 38] and pose a significant analytical challenge for conventional chemical ionisation approaches. For example, PTR-MS employing water as the reagent ion is unable to detect alkanes, particularly those containing fewer than six carbon atoms (e.g., hexane) [39–41]. However, the use of O₂⁺ as a reagent ion enables their detection, although it is associated with pronounced source fragmentation [42]. Acetone (*m/z* 59.049) was the most abundant feature, and has been consistently reported in healthy breath profiles [28]. The second most intense compound, *m/z* 171.149, had no match in the breath-specific HBDB database. Precautions must be taken when interpreting the presence of this compound as it may correspond to several chemical compounds present in the lab (3-pentanol, methyl tert-butyl ether or 2-methyl-2-butanol) forming an [M + 2ACN + H]^+^ adduct, or tetraethylurea as an [M-H]^+^ adduct. Although the level of this compound was twofold higher in breath than in ambient air, a contribution from the mouthpieces or filters used cannot be excluded.

Furthermore, the SICRIT interface may not exclusively detect volatile organic compounds, but may also enable the ionization of certain non-volatile species such as valine. This could occur through the introduction of microdroplets originating from the airway lining fluid, which may occasionally cross the filter pores and reach the ionization source. Such microdroplets can carry dissolved or suspended non-volatile constituents, including amino acids, small peptides, or other polar metabolites, that become partially desolvated and ionized upon entering the plasma or reagent gas environment. Consequently, the SICRIT source might provide access not only to volatile metabolites, but also to a fraction of semi-volatile or non-volatile compounds transported in an aerosol form, thereby broadening the range of detectable analytes compared with conventional gas-phase ionisation methods.

Demographic variables had minor but noteworthy effects on the breath volatilome. Twenty-five features differed according to sex, and six significantly correlated with fasting duration. These findings are partly consistent with previous studies reporting diet-, sex-, and physiology-related variations in breath VOCs [43–46]. For instance, pyrazine or pyrimidine (*m/z* 81.045), possibly food-derived, was more abundant in non-fasting individuals, reinforcing the importance of controlling for dietary factors in breath analysis studies.

In summary, this study presents the first comprehensive evaluation of SICRIT-HRMS for human breath analysis. The method successfully detected a broad spectrum of VOCs, demonstrated reproducibility in intra-subject VOC profiles, and identified candidate biomarkers related to breath physiology. Despite certain limitations, including flow variability, the absence of absolute quantification, and challenges in compound annotation, SICRIT-HRMS emerges as a promising and versatile platform for breathomics research. Expanding its application to disease-specific cohorts will be key to assessing its diagnostic potential and supporting the development of non-invasive tools for respiratory and systemic disease detection.

## Supplementary Information

Below is the link to the electronic supplementary material.

ESM 1 (DOCX 103 KB)

## Data Availability

The data that support the findings of this study are not openly available due to reasons of sensitivity and are available from the corresponding author upon reasonable request.
