# Supplementary material for: Soft ionization by chemical reaction in transfer—high-resolution mass spectrometry for clinical exhaled breath profiling
Source: Anal Bioanal Chem. 2026 Mar 15;418(11):3227–38. doi: 10.1007/s00216-026-06425-1 (PMC13197316; doi:10.1007/s00216-026-06425-1)
Supplement: Supplementary file 1 — (DOCX 103 KB) [file 216_2026_6425_MOESM1_ESM.docx]

**SUPPLEMENTARY INFORMATION**

**Soft ionization by chemical reaction in transfer - high resolution mass spectrometry for clinical exhaled breath profiling**

**Running title:** SICRIT-HRMS for breath analysis

Camille Roquencourt,^1,2^ Elodie Lamy,^2,3^ Nicolas Hunzinger,^2,3^ Hélène Salvator,^2,,4,5^ Philippe Devillier,^1^ Emmanuelle Bardin,^1,2,3,6^ Stanislas Grassin-Delyle^1,2,3^

^1^Hôpital Foch, Exhalomics^®^, Suresnes, France; ^2^Institut Hospitalo-Universitaire Comprehensive SEPSIS Center, Paris-Saclay University, Saclay, France; ^3^Université Paris-Saclay, UVSQ, INSERM, Infection et inflammation (2I), U1173, Département de Biotechnologie de la Santé, Unité Technologies pour la Santé et le Médicament, Montigny le Bretonneux, France; ^4^Hôpital Foch, Service de pneumologie, Suresnes, France; ^5^Université Paris-Saclay, UVSQ, UFR Simone Veil-Santé, VIM-Suresnes, UMR0892, Suresnes, France; ^6^Université Paris Cité, INSERM U1151, CNRS UMR8253, Institut Necker Enfants Malades, Paris, France.

**Corresponding author:** Stanislas Grassin-Delyle; Exhalomics, Hôpital Foch; 40, rue Worth; 92150 Suresnes; France; E-mail address: [s.grassindelyle@hopital-foch.com](mailto:s.grassindelyle@hopital-foch.com); Phone: +33.1.46.25.73.93.


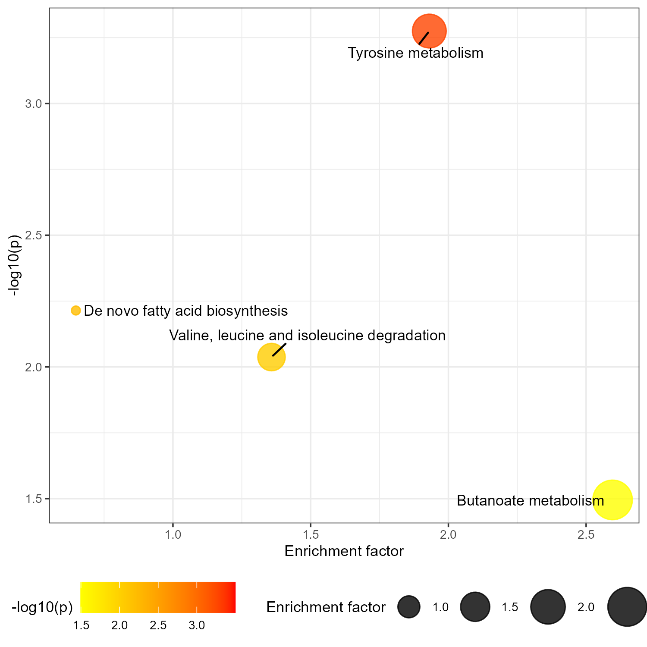


**Fig. S1:** Results of the Mummichog pathway annotation analysis, presenting identified pathways grouped according to their respective metabolic families.


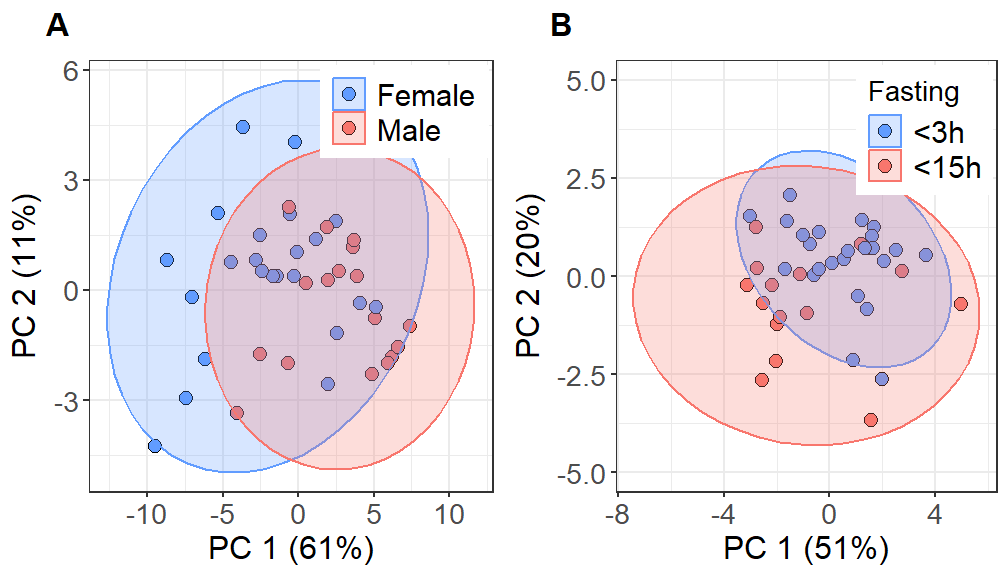


**Fig. S2:** PCA plots based on (A) the 25 VOCs associated with gender and (B) the 6 VOCs associated with fasting duration

**Table S1.** List of the 25 features significantly associated with gender, with putative compound annotations assigned according to [M+H]⁺ adducts referenced in the Human Breathomics Database (HBDB)

| *m/z* | Median log-intensity  Female Male | | p-value | Formula | IUPAC name | PubChem IDs | Previously detected in human breath |
| --- | --- | --- | --- | --- | --- | --- | --- |
| 60.081 | 13.6 | **14.5** | 0.004 | C_3_H_9_N | N,N-dimethylmethanamine/N-methylethanamine/propan-2-amine/ isopropylamine | 1146/12219 | Yes |
| 66.047 | 9.4 | **10.5** | 0.003 |  |  |  | No |
| 67.054 | 15.0 | **15.4** | <0.001 | C_5_H_6_ | Cyclopenta-1,3-diene/2-methylbut-1-en-3-yne/ pent-3-en-1-yne/(E)-pent-3-en-1-yne/2-penten-4-yne/3-penten-1-yne | 7612/62323/137090/638083 | Yes |
| 68.062 | 13.1 | **14.0** | <0.001 |  |  |  | No |
| 73.048 | **11.6** | 11.1 | 0.004 |  |  |  | No |
| 75.026 | **12.7** | 12.1 | 0.002 | C_3_H_6_S | Propylene sulfide | 14072 | Yes |
| 76.076 | 10.2 | **11.6** | 0.005 | C_3_H_9_NO | N,N-dimethylmethanamine oxide | 1145 | Yes |
| 83.069 | 9.9 | **11.1** | <0.001 |  |  |  | No |
| 85.089 | 9.3 | **11.1** | <0.001 |  |  |  | No |
| 93.037 | **12.9** | 12.2 | 0.005 | C_2_H_8_O_2_Si | Dihydroxy(dimethyl)silane | 14014 | Yes |
| 95.016 | **14.2** | 13.4 | 0.002 | C_2_H_6_O_2_S | Methylsulfonylmethane | 6213 | Yes |
| 97.052 | 9.2 | **10.2** | <0.001 |  |  |  | No |
| 99.063 | 9.4 | **10.3** | 0.003 |  |  |  | No |
| 118.086 | 10.6 | **11.5** | 0.006 | C_5_H_11_NO_2_ | Valine |  | No |
| 121.064 | **15.2** | 15.0 | 0.006 | C_8_H_8_O | 2-phenylacetaldehyde/1-phenylethanone/2-methylbenzaldehyde/4-methylbenzaldehyde | 998/7410/10722 | Yes |
| 134.081 | 8.2 | **11.0** | <0.001 |  |  |  | No |
| 136.097 | 8.3 | **11.9** | <0.001 |  |  |  | No |
| 143.143 | **13.2** | 13.1 | 0.008 | C_9_H_18_O | Nonan-2-one/nonanal/2,4-dimethylhept-1-en-4-ol/cyclooctylmethanol/1-nonen-4-ol/1-nonen-3-ol | 13187/31289/556997 | Yes |
| 152.083 | 11.2 | **12.1** | 0.008 |  |  |  | No |
| 204.159 | 11.3 | **12.2** | 0.010 |  |  |  | No |
| 218.138 | 11.8 | **12.5** | 0.006 |  |  |  | No |
| 242.176 | 11.8 | **12.6** | 0.008 |  |  |  | No |
| 244.191 | 10.6 | **12.2** | 0.006 |  |  |  | No |
| 248.223 | 7.9 | **8.9** | 0.006 |  |  |  | No |
| 294.209 | 8.8 | **11.2** | 0.006 |  |  |  | No |

**Table S1.** List of the 6 features significantly associated with fasting duration, including putative annotations based on [M+H]^+^ adducts from the Human Breathomics Database (HBDB).

| *m/z* | Median log-intensity  <3h <15h | | *p*-value | Formula | IUPAC name | PubChem IDs | Previously detected in human breath |
| --- | --- | --- | --- | --- | --- | --- | --- |
| 81.045 | **13.2** | 12.2 | 0.007 | C_4_H_4_N_2_ | pyrimidine/pyrazine | 9260 | Yes |
| 83.049 | 15.8 | **16.1** | 0.030 | C_5_H_6_O | 2-methylfuran/3-methylfuran/ cyclopent-2-en-1-one | 10797/13587/13588 | Yes |
| 84.057 | 15.2 | **15.7** | 0.050 |  |  |  | No |
| 85.065 | 15.4 | **15.8** | 0.050 | C_5_H_8_O | 3,4-dihydro-2H-pyran/ cyclopentanone/3-methylbut-3-en-2-one / pent-1-en-3-one/3-methylbut-2-enal/(E)-pent-3-en-2-one/(E)-2-methylbut-2-enal/ senecialdehyde/2-ethylacrolein/ 2- ethacrolein/3-methyl-2-butenal/3-penten-2-one, (E)- | 8080/8452/13143/15394/61020/637920/5321950/59270504 | Yes |
| 91.021 | 8.6 | **9.3** | 0.015 | C_3_H_6_OS | S-methyl ethanethioate/1-sulfanylpropan-2-one | 73750/520144 | Yes |
| 100.052 | 13.1 | **13.5** | 0.040 |  |  |  | No |
